# Supplementary material for: DAPK interacts with Patronin and the microtubule cytoskeleton in epidermal development and wound repair
Source: eLife. 2016 Sep 23;5:e15833. doi: 10.7554/eLife.15833 (PMC5053806; doi:10.7554/eLife.15833)
Supplement: Supplementary file 1. — (A) List of new strains and genotypes. (B) List of new plasmids. DOI: http://dx.doi.org/10.7554/eLife.15833.029 [file elife-15833-supp1.docx]

**Supplemental File 1A. New strains and genotypes**

| Strain | DNA construct/allele descript | New Transgene/  allele | Genetic background |
| --- | --- | --- | --- |
| CZ15506 |  |  | *dapk-1(ju4); ptrn-1(ju698)* |
| CZ17509 |  |  | *dapk-1(ju4); ptrn-1(lt1)* |
| CZ18000 |  |  | *dapk-1(ju4); PTRN-1::mcherry (ltSi183); ptrn-1(ju698)* |
| CZ18102 |  |  | *ptrn-1(ju698)* |
| CZ15505 |  |  | *dapk-1(ju4) dhc-1(ju697)* |
| CZ18060 |  |  | *dapk-1(ju4) dhc-1(or195)* |
| CZ19321 |  |  | *dapk-1(ju4) dhc-1(or195); orIs17* |
| CZ23031 |  |  | *dapk-1(gk219) dhc-1(or195)* |
| CZ18570 |  |  | *dapk-1(gk219) ptrn-1(lt1)* |
| CZ18444 |  |  | *dapk-1(ju4); klp-7(tm2143)* |
| CZ18445 |  |  | *dapk-1(ju4); klp-7(tm2143); ptrn-1(ju698)* |
| CZ18920 |  |  | *dapk-1(gk219); klp-7(tm2143)* |
| CZ19322 |  |  | *dapk-1(ju4) dhc-1(ju697); orIs17* |
| CZ21884 |  |  | *dapk-1(ju4); unc-116(e2310)* |
| CZ21866 |  |  | *dapk-1(gk219); unc-116(e2310)* |
| CZ21889 |  |  | *dapk-1(ju4); dhc-1(ju697); klp-7(tm2143)* |
| CZ18563 |  |  | *dapk-1(ju4); dylt-2 (gk762)* |
| CZ20536 |  |  | *dapk-1(ju4); dnc-1(or404)* |
| CZ18569 |  |  | *dapk-1(ju4); efa-6 (tm3124)* |
| CZ18832 |  |  | *dapk-1(ju4); mcrs-1(tm3861)* |
| CZ18829 |  |  | *dapk-1(ju4); ttll-5(tm3360)* |
| CZ19320 |  |  | *dapk-1(ju4); unc-14 (e57)* |
| CZ20542 |  |  | *dapk-1(ju4) ; unc-70(e524)* |
| CZ19908 |  |  | *dapk-1(gk219); mei-1(or642)* |
| CZ21860 |  |  | *dapk-1(gk219); spas-1(tm683)* |
| CZ21861 |  |  | *dapk-1(gk219); sma-1(e30)* |
| CZ21862 |  |  | *dapk-1(gk219); ebp-2(gk756)* |
| CZ21863 |  |  | *dapk-1(gk219); F47G4.5(ok2667)* |
| CZ21864 |  |  | *dapk-1(gk219); unc-51(ky347)* |
| CZ21865 |  |  | *dapk-1(gk219); efa-6(tm3124)* |
| CZ20883 |  |  | *dapk-1(ju4); ebp-1(tm1357)* |
| CZ21189 |  |  | *dapk-1(ju4); ebp-2(gk756)* |
| CZ21881 |  |  | *dapk-1(ju4); sma-1(e30)* |
| CZ21883 |  |  | *dapk-1(ju4); unc-51 (ky347)* |
| CZ24640 | *dapk-1(23402 bp deletion, 682 bp upstream of gene, 143 bp downstream of gene)* | *ju1936* |  |
| CZ24641 | *dapk-1 deletion (22922 bp deletion, 205 bp upstream, 140bp downstream)* | *ju1937* |  |
| CZ24642 | *dapk-1 deletion (22920 bp deletion, 202 bp upstream, 141 bp downstream)* | *ju1938* |  |
| CZ20165 | *dapk-1(C/T Q608*)* | *ju1143* | *dapk-1(ju4) o/c1* |
| CZ20166 | *dapk-1(C/T R48*)* | *ju1145* | *dapk-1(ju4) o/c1* |
| CZ19770 | *unc-33(G/A E527K)* | *ju1149* | *dapk-1(ju4) o/c2* |
| CZ19878 |  |  | *frIs7; ptrn-1(lt1)* |
| CZ19905 |  |  | *dapk-1(ju4) dhc-1(or195); fris7* |
| CZ19907 |  |  | *dapk-1(ju4) dhc-1(ju697); fris7* |
| CZ20171 |  |  | *dhc-1(or195); fris7* |
| CZ17965 |  |  | *frIs7; ptrn-1(tm5597)* |
| CZ17999 |  |  | *dapk-1(ju4); frIs7; ptrn-1(tm5597)* |
| CZ17964 |  |  | *dapk-1(ju4); frIs7; ptrn-1(ju698)* |
| CZ21890 |  |  | *dapk-1(ju4); klp-7 (tm2143); frIs7; ju698* |
| CZ20170 |  |  | *klp-7(tm2143); fris7* |
| CZ21874 |  |  | *unc-116 (e2310); frIs7* |
| CZ21886 |  |  | *dapk-1(ju4); unc-116 (e2310); frIs7* |
| CZ21878 |  |  | *frIs7; sma-1 (e30)* |
| CZ21885 |  |  | *dapk-1(ju4); frIs7; sma-1 (e30)* |
| CZ24105 |  |  | *dhc-1(or195); frIs7; ptrn-1(lt1)* |
| CZ23253 |  |  | *dapk-1(ju4) dhc-1(or195); frIs7; ptrn-1(lt1)* |
| CZ24124 | [P*dpy-7*-mKate2-PTRN-1] | *juEx7387* | *frIs7; ptrn-1(lt1)* |
| CZ24122 | [P*dpy-7*-mKate2-PTRN(CKK)] | *juEx7385* | *frIs7; ptrn-1(lt1)* |
| CZ21897 |  |  | *Pdpy-7-GFP-tbb-2 (ltSi570) dapk-1(ju4); ptrn-1(lt1)* |
| CZ21894 |  |  | *dapk-1(ju4) ltSi570* |
| CZ22742 |  |  | *ltSi570; cat-4(tm773)* |
| CZ18919 |  |  | *Pcol-19-GFP-moesin (juIs352); ptrn-1(lt1)* |
| CZ18921 |  |  | *juIs352 dapk-1(ju4); ptrn-1(lt1)* |
| CZ19904 | [P*dpy-7*-PTRN-1::GFP] | *juEx6036* | *dapk-1(ju4); ptrn-1(lt1)* |
| CZ22136 | [P*dpy-7*-GFP::PTRN-1] | *juEx6697* | *ptrn-1(lt1)* |
| CZ22725 |  | *juEx6697* | *dapk-1(ju4)* |
| CZ22726 |  | *juEx6697* | *dapk-1(ju4); ptrn-1(lt1)* |
| CZ22134 | [P*dpy-7*-GFP::CKK] | *juEx6695* | *ptrn-1(lt1)* |
| CZ22160 |  | *juEx6695* | WT |
| CZ22161 |  | *juEx6695* | *ptrn-1(ju698)* |
| CZ22162 |  | *juEx6695* | *ptrn-1(tm5597)* |
| CZ22138 | [Pdpy-7-GFP::PTRN-1(ΔCKK)] | *juEx6699* | *ptrn-1(lt1)* |
| CZ19383 | [P*dpy-7*-GFP::PTRN-1(CKK)] | *juEx5853* | *dapk-1(ju4); ptrn-1(lt1)* |
| CZ22147 | [P*dpy-7*-GFP::PTRN-1(CH)] | *juEx6704* | *dapk-1(ju4); ptrn-1(lt1)* |
| CZ22146 | [P*dpy-7*-GFP::PTRN-1(CC)] | *juEx6703* | *dapk-1(ju4); ptrn-1(lt1)* |
| CZ23024 |  | *juEx6699* | *dapk-1(ju4); ptrn-1(lt1); juEx6826* |
| CZ22257 | [P*dpy-7*-GFP::ΔCH] | *juEx6736* | *ptrn-1(lt1)* |
| CZ22273 | [Pdpy-7-GFP::PTRN-1(ΔCHCC1)] | *juEx6752* | *ptrn-1(lt1)* |
| CZ22276 | [P*dpy-7*-GFP::PTRN-1(ΔCHCC1CC2longer)] | *juEx6755* | *ptrn-1(lt1)* |
| CZ22277 | [P*dpy-7-*GFP::PTRN-1(ΔCHCC1CC2)] | *juEx6756* | *ptrn-1(lt1)* |
| CZ22280 | [P*col-19*-GFP-PTRN-1(CKK)] | *juEx6759* | *ptrn-1(lt1)* |
| CZ22465 | [P*dpy-7*-GFP::PTRN-1(ΔCC2) | *juEx6817* | *ptrn-1(lt1)* |
| CZ22466 | [P*dpy-7*-GFP::PTRN-1(ΔCC3)] | *juEx6818* | *ptrn-1(lt1)* |
| CZ22467 | [P*dpy-7*-GFP::PTRN-1(ΔCC)] | *juEx6819* | *ptrn-1(lt1)* |
| CZ23030 |  | *juEx6819* | *WT* |
| CZ22145 | [P*dpy-7*-GFP::ΔCC1] | *juEx6702* | *ptrn-1(lt1)* |
| CZ8985 | [P*dpy-7*-GFP::DAPK-1(FL)] | *juEx1774* | *dapk-1(ju4)* |
| CZ22260 | [P*dpy-7*-GFP::DAPK-1(ΔDDΔCt)] | *juEx6739* |  |
| CZ23014 |  | *juEx6739* | *dapk-1(ju4)* |
| CZ22262 | [P*dpy-7*-GFP::DAPK-1(mid+DD+Ct)] | *juEx6742* |  |
| CZ23256 |  | *juEx6742* | *dapk-1(ju469)* |
| CZ23257 |  | *juEx6742* | *dapk-1(ju4)* |
| CZ22265 | [P*dpy-7-*GFP::DAPK-1(ΔKinase)] | *juEx6744* |  |
| CZ23012 |  | *juEx6744* | *dapk-1(ju469)* |
| CZ23061 |  | *juEx6744* | *dapk-1(ju4)* |
| CZ22269 | [P*dpy-7-*GFP::DAPK-1(ΔCalmB)] | *juEx6748* |  |
| CZ23011 |  | *juEx6748* | *dapk-1(ju469)* |
| Unable to freeze |  | *juEx6748* | *dapk-1(ju4)* |
| CZ22476 | [P*dpy-7*-GFP::DAPK-1(CalmB only)] | *juEx6828* |  |
| CZ22267 | [Pdpy-7-GFP::DAPK-1(ΔcytoskB)] | *juEx6746* |  |
| CZ23013 |  | *juEx6746* | *dapk-1(ju4)* |
| CZ22723 | [P*dpy-7*-GFP::DAPK-1(cytoskB)] | *juEx6924* |  |
| CZ23254 |  | *juEx6924* | *dapk-1(ju469)* |
| CZ23255 |  | *juEx6924* | *dapk-1(ju4)* |
| CZ22482 | [P*dpy-7*-GFP::DAPK-1(ANK only)] | *juEx6834* |  |
| CZ22717 | [*Pdpy-7*-GFP::DAPK-1(Kinase only)] | *juEx6918* |  |
| CZ23019 | [P*dpy-7*-GFP::DAPK-1(K57A)] | *juEx7011* |  |
| CZ23025 |  | *juEx7011* | *dapk-1(ju4)* |
| CZ23022 | [P*dpy-7-*GFP::DAPK-1(S179L)] | *juEx7013* |  |
| CZ16503 | [P*col-19*-GFP::DAPK-1] | *juEx4781* | *WT* |
| CZ22123 |  | *juEx4781* | *dhc-1(or195)* |
| CZ17886 |  | *juEx4781* | *dapk-1(ju4)* |
| CZ18915 |  | *juEx4781* | *ptrn-1(lt1)* |
| CZ22727 |  | *juEx4781* | *cat-4(tm773)* |
| CZ22291 |  | *juEx4781* | *unc-116(e2310)* |
| CZ9336 | [P*dpy-7*-GFP::RAB-5] | *juEx1921* | WT |
| CZ21789 | [P*col-19*-GFP::TBB-2] | *juSi239* |  |
| CZ23247 |  | *juSi239* | *dapk-1(ju4) ptrn-1(lt1)* |
| CZ23248 |  | *juSi239* | *dapk-1(ju4)* |
| CZ23249 |  | *juSi239* | *ptrn-1(lt1)* |
| CZ24779 |  | *juSi239* | *dapk-1(gk219)* |
| CZ24778 |  | *juSi239* | *dapk-1(gk219) ptrn-1(lt1)* |
| CZ23018 | [P*dpy-7*-PTRN-1::GFP] | *ltSi541* | *dapk-1(ju4); ptrn-1(lt1)* |
| CZ22739 | [P*dpy-7*-mKate2::PTRN-1] | *juEx6820* | *ltSi570; ptrn-1(lt1)* |
| CZ23015 | [P*dpy-7*-mKate2::PTRN-1(CKK)] | *juEx6826* | *ltSi570; ptrn-1(lt1)* |
| CZ22736 | [P*dpy-7*-mKate2::PTRN-1(CC) | *juEx6822* | *ltSi570* |
| CZ22814 |  | *juEx6820* | *ptrn-1(lt1); juEx1774* |
| CZ14453 | [P*col-19*-EBP-2::GFP] | *juEx3762* | *WT* |
| CZ18902 |  | *juEx3762* | *ptrn-1(lt1)* |
| CZ18903 |  | *juEx3762* | *dapk-1(ju4)* |
| CZ24151 |  | *juEx3762* | *dapk-1(ju4); ptrn-1(lt1)* |

All plasmids with *dpy-7* or *col-19* promoter were injected at 1 ng/μl unless stated otherwise.

**Supplemental File 1B. New plasmids**

| **Name** | **Plasmid #** | **Construction** |  |
| --- | --- | --- | --- |
| P*dpy-7*-GFP::PTRN-1 | pCZGY2467 | LR recombination with pCZGY2409 (PTRN-1 +stop pCR8) and pCZGY2096 (P*dpy-7*-GFP-gw) | |
| P*dpy-7*-PTRN-1 | pCZGY2468 | LR recombination with pCZGY2409 (PTRN-1 +stop pCR8) and pCZGY146 (P*dpy-7*-gw) | |
| P*dpy-7*-PTRN-1::GFP | pCZGY2469 | LR recombination with pCZGY2410 (PTRN-1 -stop pCR8) and pCZGY1598 (P*dpy-7*-gw-GFP) | |
| P*dpy-7*-GFP::PTRN-1(CH) | pCZGY2452 | LR recombination with pCZGY2411 (PTRN-1(CH) pCR8) and pCZGY2096 (P*dpy-7*-GFP-gw) | |
| P*dpy-7*-GFP:: PTRN-1(CKK) | pCZGY2453 | LR recombination with pCZGY2413 (PTRN-1(CKK) pCR8) and pCZGY2096 | |
| P*dpy-7*-GFP::PTRN-1(ΔCH) | pCZGY2454 | LR recombination with pCZGY2414 (PTRN-1(ΔCH) pCR8) and pCZGY2096 (P*dpy-7*-GFP-gw) | |
| P*dpy-7*-GFP:: PTRN-1(ΔCKK) | pCZGY2455 | LR recombination with pCZGY2415 (PTRN-1(ΔCKK) pCR8) and pCZGY2096 (P*dpy-7*-GFP-gw) | |
| P*dpy-7*-GFP::PTRN-1(CC) | pCZGY2456 | LR recombination with pCZGY2412 (PTRN-1(CC) pCR8) and pCZGY2096 (P*dpy-7*-GFP-gw). aa301-969. | |
| P*dpy-7*-GFP::PTRN-1(ΔCC1) | pCZGY2466 | LR recombination with pCZGY2442 (PTRN-1(ΔCC1)) and pCZGY2096 (P*dpy-7*-GFP-gw). Delete aa 402-452. | |
| P*dpy-7*-GFP::PTRN-1(ΔCHCC1) | pCZGY2778 | Gibson Cloning. Template: pCZGY246. Delete aa 1-452. | |
| P*dpy-7*-GFP::PTRN-1(ΔCHCC1CC2longer) | pCZGY2779 | Gibson Cloning. Template: pCZGY246. Delete aa 1-615. | |
| P*dpy-7*-GFP::PTRN-1(ΔCHCC1CC2) | pCZGY2780 | Gibson Cloning. Template: pCZGY246. Delete aa 1-777. | |
| P*dpy-7*-GFP::PTRN-1(ΔCC2) | pCZGY2781 | Gibson Cloning. Template: pCZGY246. Delete aa 473-615. | |
| P*dpy-7*-GFP::PTRN-1(ΔCC3) | pCZGY2782 | Gibson Cloning. Template: pCZGY246. Delete aa 786-969. | |
| P*dpy-7*-GFP::PTRN-1(ΔCC) | pCZGY2783 | Gibson Cloning. Template: pCZGY246. Delete aa 301-947. | |
| P*col-19*-GFP::PTRN-1(CKK) | pCZGY2448 | LR recombination with pCZGY2413 (PTRN-1(CKK) pCR8) and pCZGY2097 (P*col-19*-GFP-gw) | |
| DAPK-1(ΔDDΔCt)pCR8 | pCZGY2784 | Gibson Cloning. Template: pCZGY440 (DAPK-1 pCR8). Delete aa 1300-1426. | |
| DAPK-1(mid+DD+Ct) pCR8 | pCZGY2785 | Gibson Cloning. Template: pCZGY440 (DAPK-1 pCR8). aa 845-1426. | |
| DAPK-1(ΔKinase) pCR8 | pCZGY2786 | Gibson Cloning. Template: pCZGY440 (DAPK-1 pCR8). Delete aa 1-275 | |
| DAPK-1(ΔcytoskB) pCR8 | pCZGY2787 | Gibson Cloning. Template: pCZGY440 (DAPK-1 pCR8). Delete aa 649-844. | |
| DAPK-1(ΔCalmB)] pCR8 | pCZGY2788 | Gibson Cloning. Template: pCZGY440 (DAPK-1 pCR8). Delete aa 288-320. | |
| DAPK-1(Kin+CalmB) pCR8 | pCZGY2789 | Gibson Cloning. Template: pCZGY440 (DAPK-1 pCR8). aa 1-320. | |
| DAPK-1(ANK only) pCR8 | pCZGY2790 | Gibson Cloning. Template: pCZGY440 (DAPK-1 pCR8). aa 373-637. | |
| DAPK-1(cytoskB) pCR8 | pCZGY2791 | Gibson Cloning. Template: pCZGY440 (DAPK-1 pCR8). aa 649-844. | |
| DAPK-1(Kinase only) pCR8 | pCZGY2792 | Gibson Cloning. Template: pCZGY440 (DAPK-1 pCR8). aa 1-275. | |
| P*dpy-7*-GFP-DAPK-1(ΔDDΔCt) | pCZGY2793 | LR recombination with pCZGY2784 (DAPK-1(ΔDDΔCt) pCR8) and pCZGY2096 (P*dpy-7*-GFP-gw) | |
| P*dpy-7*-GFP-DAPK-1(mid+DD+Ct) | pCZGY2794 | LR recombination with pCZGY2785 (DAPK-1(mid+DD+Ct pCR8) and pCZGY2096 (P*dpy-7*-GFP-gw) | |
| P*dpy-7*-GFP-DAPK-1(ΔKinase) | pCZGY2795 | LR recombination with pCZGY2786 (DAPK-1(Δkinase pCR8) and pCZGY2096 (P*dpy-7*-GFP-gw) | |
| P*dpy-7*-GFP-DAPK-1(ΔcytoskB) | pCZGY2796 | LR recombination with pCZGY2787 (DAPK-1(ΔcytoskB) pCR8) and pCZGY2096 (P*dpy-7*-GFP-gw) | |
| P*dpy-7*-GFP-DAPK-1(ΔCaMBD) | pCZGY2797 | LR recombination with pCZGY2788 (DAPK-1(ΔCaMBD) pCR8) and pCZGY2096 (P*dpy-7*-GFP-gw) | |
| P*dpy-7*-GFP-DAPK-1(Kin+CaMBD) | pCZGY2798 | LR recombination with pCZGY2789 (DAPK-1(Kin+CaMBD pCR8) and pCZGY2096 (P*dpy-7*-GFP-gw) | |
| P*dpy-7*-GFP-DAPK-1(ANK only) | pCZGY2799 | LR recombination with pCZGY2790 (DAPK-1(ANK pCR8) and pCZGY2096 (P*dpy-7*-GFP-gw) | |
| P*dpy-7*-GFP-DAPK-1(cytoskB) | pCZGY2800 | LR recombination with pCZGY2791 (DAPK-1(cytoskBD) pCR8) and pCZGY2096 (P*dpy-7*-GFP-gw) | |
| P*dpy-7*-GFP-DAPK-1(Kinase only) | pCZGY2801 | LR recombination with pCZGY2792 (DAPK-1(kinase) pCR8) and pCZGY2096 (P*dpy-7*-GFP-gw) | |
| P*dpy-7*-GFP::DAPK-1(K57A) | pCZGY2802 | Gibson cloning. Template: pCZGY501 (P*dpy-7*-GFP-DAPK-1). | |
| P*dpy-7*-GFP::DAPK-1(S179L) | pCZGY2803 | LR recombination with (DAPK-1(S179L) pCR8) and pCZGY2096 (P*dpy-7*-GFP-gw) | |
| P*dpy-7*-mKate2::PTRN-1 | pCZGY2804 | Gibson Cloning. Template: pCZGY246. | |
| P*dpy-7*-mKate2::PTRN-1(CC) | pCZGY2805 | Gibson Cloning. Template: pCZGY246. | |
| P*dpy-7*-mKate2::PTRN-1(CKK) | pCZGY2806 | Gibson Cloning. Template: pCZGY246. | |
| PCMV-Flag-DAPK-1 | pCZGY3111 | LR reaction with pCZGY440 (DAPK-1 cDNA pCR8) and pCZGY57 (PCMV-Flag-gw). | |
| PCMV-HA-PTRN-1 | pCZGY3112 | LR reaction with pCZGY2409 (PTRN-1 cDNA pCR8) and pCZGY58 (PCMV-HA-gw). | |
